# Identification of *C*-glycosyl flavones by high performance liquid chromatography electrospray ionization mass spectrometry and quantification of five main *C*-glycosyl flavones in *Flickingeria fimbriata*

**DOI:** 10.1186/s13065-019-0616-5

**Published:** 2019-07-23

**Authors:** Yawen Wang, Zhiyun Liang, Xian Liao, Chujuan Zhou, Zhenshan Xie, Sha Zhu, Gang Wei, Yuechun Huang

**Affiliations:** 1 College of the First Clinical Medical, Guangzhou University of Chinese Medicine, Guangzhou, 510405 China; 2 The First Affiliated Hospital of Guangzhou University of Chinese Medicine, Guangzhou, 510405 China; 3 School of Pharmaceutical Science, Guangzhou University of Chinese Medicine, Guangzhou, 510006 China; 4 Shaoguan Institute of Danxia Dendrobium Officinale (SIDDO), Shaoguan, 512005 China

**Keywords:** *Flickingeria fimbriata*, Acylated *C*-glycosyl flavones, Flavonoids, Multiple stage tandem mass spectrometry, High-performance liquid chromatography, Isomers, Quantitative analysis

## Abstract

*Flickingeria fimbriata* is commonly applied in China as a traditional Chinese medicine (TCM), however the quality control of it is incomplete. In this work, we aim to identify and quantify the structures of *C*-glycosyl flavones in *F. fimbriata*. High performance liquid chromatography-diode array detector (HPLC-DAD) and High performance liquid chromatography–electrospray ionization–multiple stage tandem mass spectrometry (HPLC–ESI–MS^n^) methods were combined to identify *C*-glycosyl flavones and determine their contents. Twenty acylated *C*-glycosyl flavones and ten non-acylated *C*-glycosyl flavones were identified for the first time in *F. fimbriata* on systematic MS^n^ analysis via HPLC–ESI–MS^n^. The aglycones of all of these compounds were apigenin or chrysoeriol and were acylated with *p*-coumaric, ferulic, 3,4-dimethoxycinnamic or 3,4,5-trimethoxycinnamic acids. Furthermore, the quantification result suggest that two *C*-glycosyl flavones (vicenin-I and vicenin-III) with relative high contents were revealed to be more strongly acylated in *F. fimbriata*. The method is sufficiently precise, accurate, and sensitive for the qualitative and quantitative analysis of *C*-glycosyl flavones, which is expected to establish a standard for quality control and identification in this plant.

## Introduction

*Flickingeria fimbriata* (Bl.) Hawkes is commonly used as a source of a valuable TCM called “Shihu”, which is normal referred to *Dendrobium* genus such as *Dendrobium officinale*. And this medicinal plant is commonly used as “Shihu” in Guangdong, Guangxi and Hainan provinces. The main places of production of *F. fimbriata* were Guangdong, Guangxi and Sichuan provinces. Its efficacy in soothing lung irritation and relieving cough has been reported in Guangdong Chinese Materia Medicine Standards, which are exploited in the treatment of diseases including pneumonia, tuberculosis, bronchitis, asthma, and pleurisy [1]. Previous phytochemical studies on *F. fimbriata* mainly lied in the isolation and analysis of diterpenoids [2, 3], phenanthrenes [4, 5], sterols [6] and phenolic constituents [5]. However, quality control study in this medicinal plants is incomplete. Only trait morphological identification and microscopic identification methods were mentioned in Guangdong Chinese Materia Medicine Standards of this plants and it have no specific method to control quality. The previous study of *F. fimbriata* in our laboratory showed that 7–8 stable common peaks of flavonoids in the characteristic spectra were found using HPLC [7], five of which were characterized as non-acylated *C*-glycosyl flavones (vicenin-II, vicenin-I, schaftoside, isoschaftoside and vicenin-III) by ion-trap mass spectrometer. The other uncharacterized peaks still required further investigation to complete a quality control study. It was proved that flavone is a suitable compound for quality control study of *F. fimbriata.*

Flavonoids, as common and widespread secondary plant metabolites, distributed in all parts of plants. They present as glycosides in the vacuoles, leaves, stem, and roots of flowers [8]. Sugar substitution on the flavonoid skeleton may occur through hydroxyl groups, in the case of *O*-glycosides (*O*-glycosyl flavones), or directly to carbon atoms in the A ring in *C*-glycosides (*C*-glycosyl flavones) [9]. Contents and types of *C*-glycosyl flavones were ideal index for identifying plants from the same species for its high specificity [10]. Generally, the flavonoids classification depends on the nature of aglycones, sugars, and acylate groups. Some secondary plant metabolites occur in the form of acylated glycosyl flavones with benzoic acid and/or cinnamic acid moiety. The cinnamoyl groups including *p*-coumaroyl, feruloyl, 3,4-dimethoxycinnamoyl and 3,4,5-trimethoxycinnamoyl [11–13], and their differences lie in the number and/or position of hydroxy and methoxy substituents. Many compounds like diterpenoids and phenanthrenes in *F. fimbriata* are acylated with aromatic acids, such as trans-cinnamoyl acid [14] and methoxybenzoyl acid derivatives [3, 5, 14, 15], suggesting that the aromatic acids could be synthesized in this plants. Moreover, *O*-methyltransferase (OMT) genes revealed the internal relations of cinnamic acids with different substituents, it was possible that OMTs might be associated with the formation of 3, 4-dimethoxycinnamate and 3, 4, 5-trimethoxycinnamate in biosynthesis of plants [16]. Additionally, acylated flavonoids have several health beneficial effects including anti-inflammatory [17, 18] and antioxidant activity [19], and the acylation position on glucose is regard as a potential approach for the antioxidant and cytoprotective effects of flavonoid glycosides [20].

MS is important due to its applicability for analyzing herbal medicines. The application of electrospray ionization (ESI) enabled the analysis of flavonoid glycosides without derivatization [21]. Although distinction between glycosidic and aromatic acidic substituents of flavonoids is problematic, such as deoxyhexoside and coumaric acid, both of which lose a fragment of 146 Da [9], high-performance liquid chromatography (HPLC) combined with a diode array detector (DAD) could provide online UV spectrum for each individual peak in a chromatogram which displays different spectrums between the glycosidic and aromatic acid substituents of flavonoids. Additionally, HPLC–ESI–MS^n^, equipped with an ion trap (IT) mass analyzer can obtain a large number of fragmentation patterns and typical losses up to MS^4^ [22] which could be used to identify many complex isomers of *C*-glycoside flavones [23]. By this way, the nature of aglycones and sugars as well as the position of sugar and acyl groups could be deduced in *C*-glycoside flavones. The determination by MS fragmentation of acylated *O*-glycosyl flavones is possible [24], and the *O*-glycosylation at 2′′ and at 6′′ positions could be deduced from it [25]. However, few systematic analysis of acylated *C*-glycosyl flavones in plants via HPLC–ESI/MS^n^ combining with HPLC-DAD method was afforded before this study.

To date, 20 acylated *C*-glycosyl flavones and 10 non-acylated *C*-glycosyl flavones were identified by HPLC–ESI–MS^n^ and HPLC-DAD. These 30 compounds have not been reported yet in *F. fimbriata*. In addition, 12 batches of *F. fimbriata* were successfully quantitatively analyzed, which is expected to establish a standard for quality control and identification.

## Materials and methods

### Materials

Apigenin-6,8-di-*C*-β-d-glucoside (vicenin-2) and apigenin-6-*C*-β-d-xyloside-8-*C*-β-d-glucoside (vicenin-1) were isolated from the leaves of *D. officinale*, and these compounds were identified by comparing their UV, IR, HPLC–MS and NMR data with those in published reports [10], and purity was determined to be higher than 98% by the normalization of the peak area with HPLC. Apigenin-6-*C*-β-d-glucoside-8-*C*-β-d-xyloside (vicenin-3) was purchased from Shanghai Standard Technology Co., Ltd. (Shanghai, China), and its purity was over 98%; schaftoside 92.5% was purchased from the National Institutes for Food and Drug Control (Beijing, China). Isoschaftoside was purchased from Extrasynthese (Genay, France), and its purity was over 95%. HPLC-grade acetonitrile was purchased from Merck (Darmstadt, Germany). Analytical grade methanol, formic acid, and phosphoric acid were obtained from Fuyu Fine Chemical Co., Ltd. (Tianjin, China). Distilled water was purchased from A.S, Watson Group Co., Ltd. (Hongkong, China). YUHUA SHZ-D (III) was purchased from YUHUA Instrument Co., Ltd. (Gongyi, China).

Twelve samples of natural medicinal parts of *F. fimbriata* were collected from different regions of China (Guangdong, Guangxi, and Sichuan provinces). Of these, 6 batches were from Guangdong province (No. FF1–FF6), 4 batches were from Guangxi province (No. FF7–FF10), and 2 batches were from Sichuan province (No. FF11–FF12) (Table 1). The tested samples of *F. fimbriata* (12 batches) were authenticated by professor Yuechun Huang from The First Affiliated Hospital of Guangzhou University of Chinese Medicine, Guangzhou, China. The voucher specimens (No. FF20190701) were deposited in the School of Pharmaceutical Science, Guangzhou University of Chinese Medicine, Guangzhou.

**Table 1:** The source of *Flickingeria fimbriata* (Bl.) Hawkes

No.	Origin	Source	Collect time
FF1	Guangdong Hexiang Pharmaceutical co., LTD	Guangdong	2014.8.14
FF2	Guangdong Lifeng Pharmaceutical co., LTD	Guangdong	2014.6.9
FF3	LBX Pharmaceutical co., LTD	Guangdong	2014.6.9
FF4	Jianmin Pharmaceutical co., LTD	Guangdong	2014.6.9
FF5	Jihetang Pharmaceutical co., LTD	Guangdong	2014.6.10
FF6	Yuqingtang Pharmaceutical co., LTD	Guangdong	2014.6.10
FF7	Caizhilin Pharmaceutical co., LTD	Guangxi	2014.6.9
FF8	Dashenlin Pharmaceutical co., LTD	Guangxi	2014.8.14
FF9	Guozilin Pharmaceutical co., LTD	Guangxi	2014.8.16
FF10	Baiyuantang Pharmaceutical co., LTD	Guangxi	2014.8.16
FF11	Jihetang Pharmaceutical co., LTD	Sichuan	2014.6.9
FF12	Baohetang Pharmaceutical co., LTD	Sichuan	2014.8.16

### Preparation of standard solutions

The standard samples of vicenin-II, vicenin-I, schaftoside, isoschaftoside and vicenin-III were accurately weighted and then dissolved with methanol to produce concentrations of 121.2, 120.0, 121.6, 118.4, and 122.0 μg/mL, respectively. A mixed standard solution was prepared by mixing the standard solution with methanol to obtain a certain injection amount in the range of 0.030–1.782 μg, 0.029–3.234 μg, 0.028–1.687 μg, 0.028–1.687 μg, and 0.030–3.288 μg, respectively.

### Preparation of sample extraction

The air-dried and smashed *F. fimbriata* (0.5 g) samples from each batch material were accurately weighted and extracted with 50 mL of methanol, after being weighted with a vessel, in the case of the volatilization of methanol then refluxed for 4 h at 90 °C using a Jie Rui Er HH-4 constant temperature water bath (Jiang Su Jie Rui Er electric Co., Ltd., Jiang Su, China). The extractions were removed and cooled down. The extraction was weighted again, and methanol was added into the vessel to compensate for the lost weight. Of the filtrated extraction, 25 mL was accurately transferred into an evaporation pan. The resultant concentrated extractions were transferred to a 2 mL volumetric flask and diluted to the indicated volume (2 mL). The obtained extract was filtrated through a 0.22 μm pore-size nylon filter for MS analysis and 0.45 μm pore-size nylon filters for quantitative analysis.

### HPLC–ESI–MS^n^ and HPLC-DAD analysis condition

Analysis was performed on an HPLC system equipped with a vacuum degasser, quaternary pump, auto-sampler, and ultraviolet detector (Thermo Separation Products Inc., Riviera Beach, FL, USA) and coupled with a Thermo Finnigan LCQ FLEET (Thermo Finnigan, Riviera Beach FL, USA) ion trap mass spectrometer, equipped with an electrospray ionization interface in negative ion mode. Chromatographic separations were carried out on a Kromasil 100-5 C_18_ column (250 mm × 4.6 mm, 5 μm, Akzo Noble, Sweden), maintained at 35 °C. The mobile phases were acetonitrile (A) and 0.1% (*v/v*) formic acid (B), at a flow rate of 0.8 mL/min. The gradient elution program was 0–10 min, 14% A; 10–20 min, 14–16% A; 20–45 min, 16–22% A; and 45–80 min, 22–40% A, with an elution gradient. The injection volume was 5 μL each time. The detection wavelength was set to 340 nm. The optimized MS conditions were as follows: full-scan mode between *m/z* 50 and 1000, spray voltage 3.0 kV, capillary voltage fixed at − 35.0 V, capillary temperature 350 °C, sheath gas flow rate of 30 (arbitrary units), and auxiliary gas flow rate of 10 (arbitrary units). The data acquisition and the system control were performed using a Finnigan Xcalibur 2.0 advanced chromatography workstation (Thermo Quest Corporation, San Jose, CA, USA). HPLC-DAD analysis was performed using an Agilent 1100 system (Agilent, USA). The conditions were as same as the chromatographic separation method in HPLC–ESI–MS^n^.

### HPLC quantitative analysis condition

Quantitative analysis was performed using an Agilent 1100 system (Agilent, USA). Chromatographic separations were carried out on a Zorbax SB-Aq column (250 mm × 4.6 mm, 5 μm; Agilent, USA), maintained at 35 °C. The mobile phases were acetonitrile (A) and 0.1% (*v/v*) phosphoric acid (B) (with a gradient elution program of 0–15 min, 12% A; 15–25 min, 13–14% A; 25–30 min, 14% A; 30–40 min, 14%–12% A), at a flow rate of 0.8 mL/min. The injection volume was 5–20 μL each time. The detection wavelength was set to 340 nm.

## Results and discussion

### Identification of chemical compounds

**Fig. 1:**
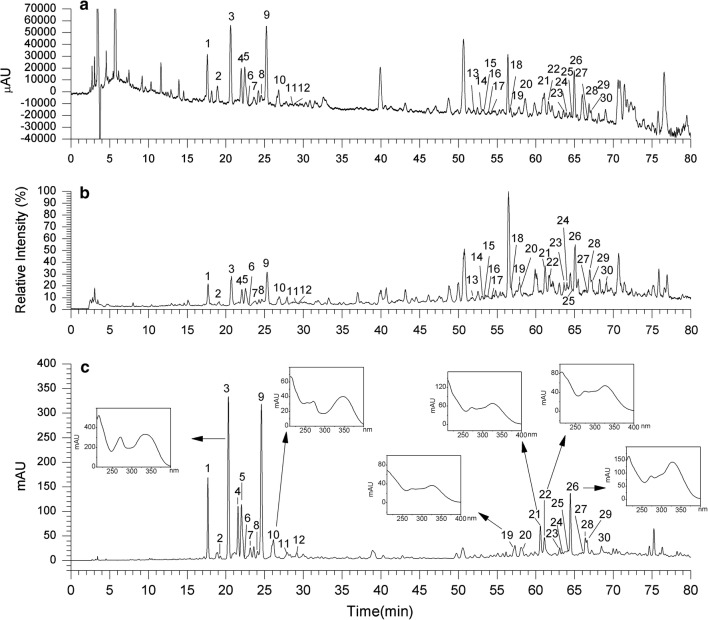
HPLC–ESI–MS^n^ analysis in the extracts of *Flickingeria fimbriata*: **a** HPLC–ESI–MS chromatogram at 340 nm; **b** HPLC–ESI–MS total ion current (TIC) profile in negative mode; **c** HPLC-DAD profile (340 nm) and UV spectra of compound 3, 10, 19, 21,22 and 26

The extract of *F. fimbriata* was analyzed by HPLC–ESI–MS^n^ and HPLC-DAD. The UV chromatogram at 340 nm is shown in Fig. 1a, and its total ion chromatograms (TICs) are shown in Fig. 1b. Flavonoids typically exhibit two major absorption bands in the ultraviolet region: Band I in the 320–385 nm region, representing B-ring absorption, and Band II in the 250–285 nm range, representing A-ring absorption [25]. These UV data are in accordance with *C*-glycosyl apigenin and chrysoeriol, respectively [11, 26]. The majority of flavonoids with cinnamoyl acid have a UV spectrum with an intense Band I at approximately 330 nm and a small Band II at approximately 270 nm (Fig. 1c), as a result of the overlapped UV spectra [27].

*C*-glycosyl flavones, with the characteristic saccharides substitution directly attached to aglycone in ring A through a C–C bond, all show substituents in position 6 (C-6) and/or 8 (C-8) of the aglycone moiety [8]. Apart from glucose, monosaccharides including xylose, arabinose, and rhamnose are ubiquitous in plants [28]. Due to the cross-ring cleavages of the flavonoid saccharide residue, characteristic ions [Ag-H+42]^−^ and [Ag-H+72]^−^ were observed in the MS^4^ spectra for the mono-*C*-glycosyl flavones, and characteristic ions [Ag-H+84]^−^ and [Ag-H+114]^−^ were observed in the MS^4^ spectra for the 6,8-di-*C*-glycosyl flavones (Ag = 270 for apigenin, Ag = 300 for chrysoeriol) [24, 29]. In *F. fimbriata*, combining the loss of mass with the previously reported results, and considering the high contents of vicenin-II, vicenin-I, schaftoside, isoschaftoside and vicenin-III, the xylose, arabinose, and glucose moieties were found to be involved in glycosylation. The major fragmentation pathways concern the cross-ring cleavages of the saccharide residue and the loss of water molecules. In negative mode, the characteristic ions of sugars in *C*-glycosyl flavones lost 120 Da and 90 Da in the hexose substituents, and 90 Da and 60 Da in pentose substituents by crossing cleavages, respectively [30].

**Fig. 2:**
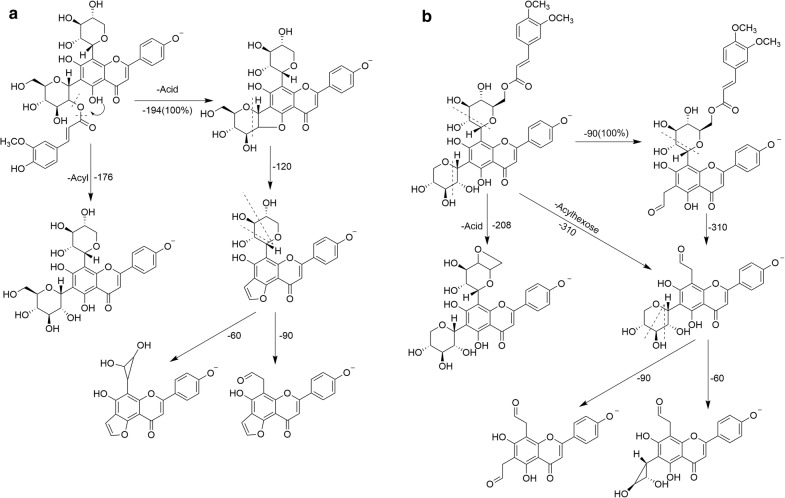
The proposed fragmentation pathways of acylated- *C*-glycosyl flavones when the position of acylation was on the hydroxyl in position 2″ and 6″ in sugar (2″-*O* and 6″-*O*), **a** Apigenin-6-*C*-(2″-*O*-feruloyl)-β-d-glucoside-8-*C*-β-d-xyloside (14) and **b** Apigenin-6-*C*-β-d-xyloside-8-*C*-(6″-*O*-3,4-dimethoxycinnamoyl)-β-d-glucoside (26)

In di-*C*-glycosyl flavones, sugar residues of different masses can be located, since the 6-*C*-sugar residue shows greater fragmentation than the 8-*C*-sugar residue [8]. From these findings, we deduced the types and position of sugars. For the acylated-*C*-glycosyl flavones, and if the base peak ions are made of [M-H-120]^−^ or [M-H-90]^−^ in MS^2^ spectra, there can be no acyl on the 6-*C*-sugar. If the base peak ions in MS^2^ spectra are constitutive of the loss in acyl-relative ([M-H-Acyl]^−^) or acid-relative ([M-H-Acid]^−^) neutral moiety, this suggests an acylation on the 6-*C*-sugar. When the position of acylation on the hydroxyl in position 2″ in sugar (2″-*O*), ions [(Ag-H+42)-18]^−^ were observed in the MS^3^ spectra for the mono-*C*-glycosyl flavones, ions [(Ag-H+84)-18]^−^ and [(Ag-H+114)-18]^−^ were observed for the di-*C*-glycosyl flavones, which differ from the position set to 6″ (6″-*O*). When hydroxyl in position 6″ in sugar (6″-*O*), ions [Ag-H+84]^−^ and [Ag-H+114]^−^ were obtained in the MS^3^ spectra from di-*C*-glycosyl flavones (Fig. 2).

**Fig. 3:**
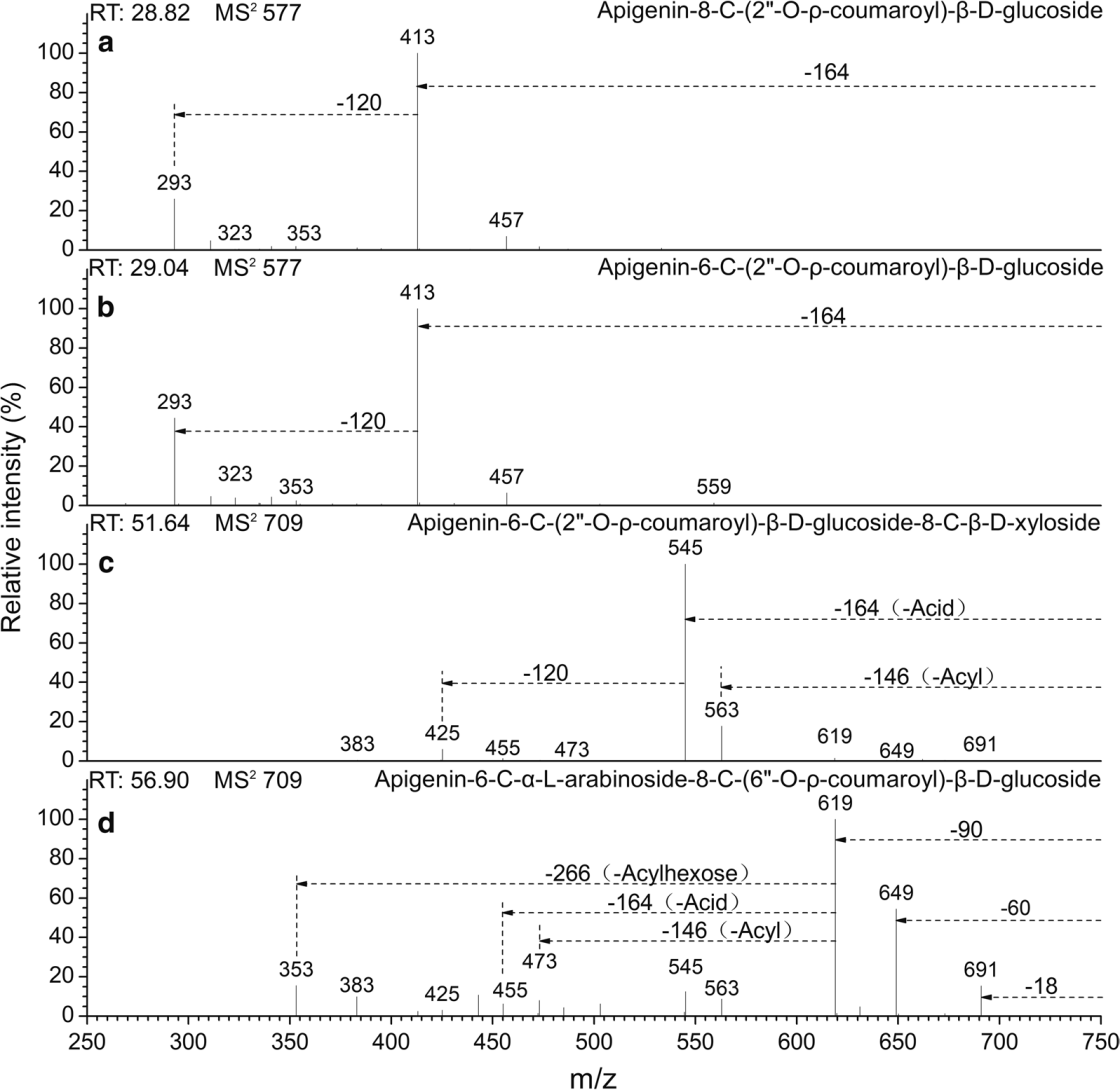
CID MS^2^ spectra fragmentations of two pairs of isomeric *C*-glycosyl flavones, acylated with *ρ*-coumaroyl from the extracts of *Flickingeria fimbriata* in negative ionization mode: **a** Apigenin-8-*C*-(2″-*O*-*ρ*-coumaroyl)-β-d-glucoside(11), **b** Apigenin-6-*C*-(2″-*O*-*ρ*-coumaroyl)-β-d-glucoside (12), **c** Apigenin-6-*C*-(2″-*O*-*ρ*-coumaroyl)-β-d-glucoside-8-*C*-β-d-xyloside (13), and **d** Apigenin-6-*C*-α-l-arabinoside-8-*C*-(6″-*O*-*ρ*-coumaroyl)-β-d-glucoside (18)

**Fig. 4:**
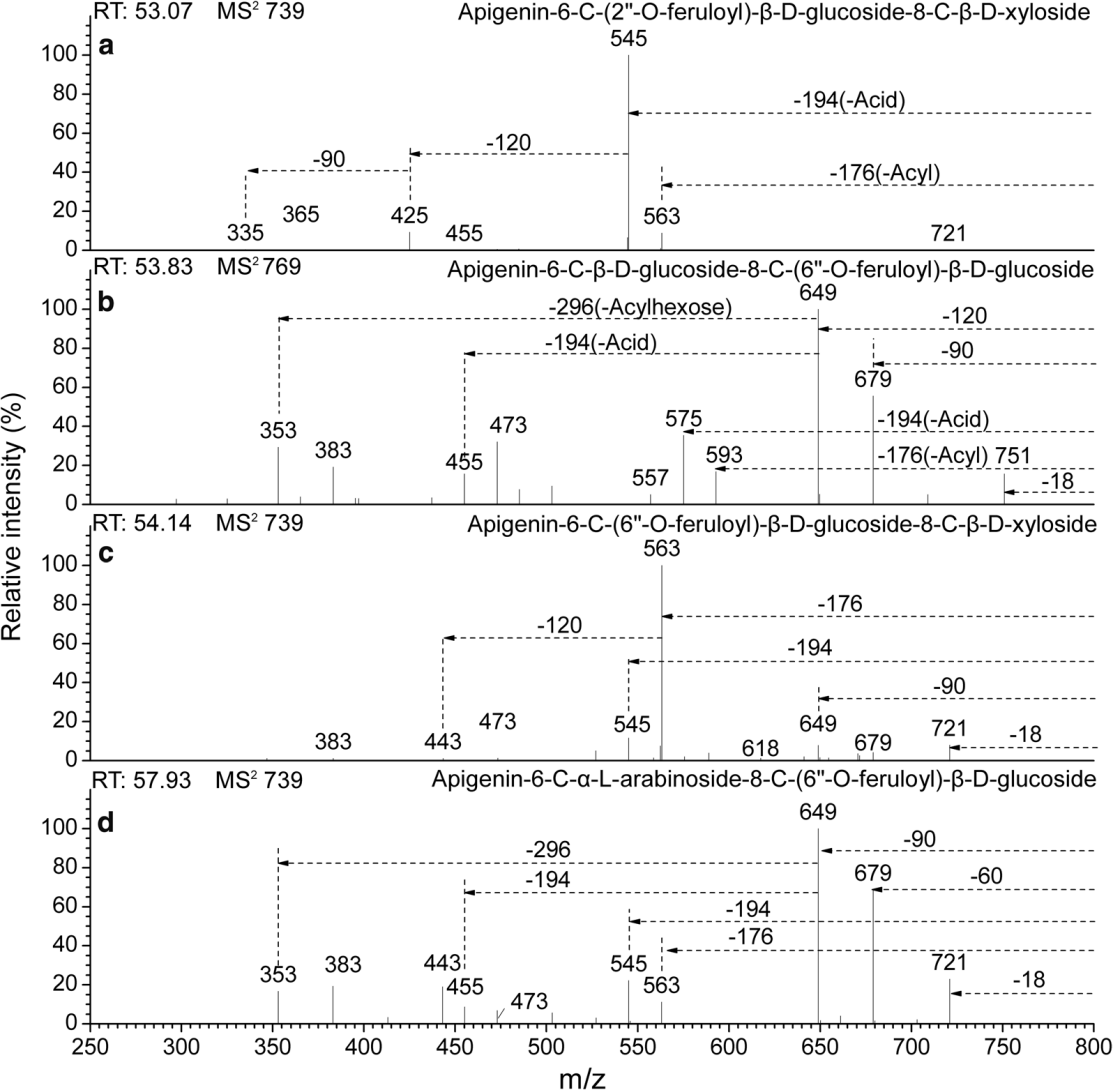
CID MS^2^ spectra fragmentations of four *C*-glycosyl flavones, acylated with feruloyl from the extracts of *Flickingeria fimbriata* in negative ionization mode: **a** Apigenin-6-*C*-(2″-*O*-feruloyl)-β-d-glucoside-8-*C*-β-d-xyloside (14), **b** Apigenin-6-*C*-β-d-glucoside-8-*C*-(6″-*O*-feruloyl)-β-d-glucoside (16), **c** Apigenin-6-*C*-(6″-*O*-feruloyl)-β-d-glucoside-8-*C*-β-d-xyloside (17), and **d** Apigenin-6-*C*-α-l-arabinoside-8-*C*-(6″-*O*-feruloyl)-β-d-glucoside (20)

**Fig. 5:**
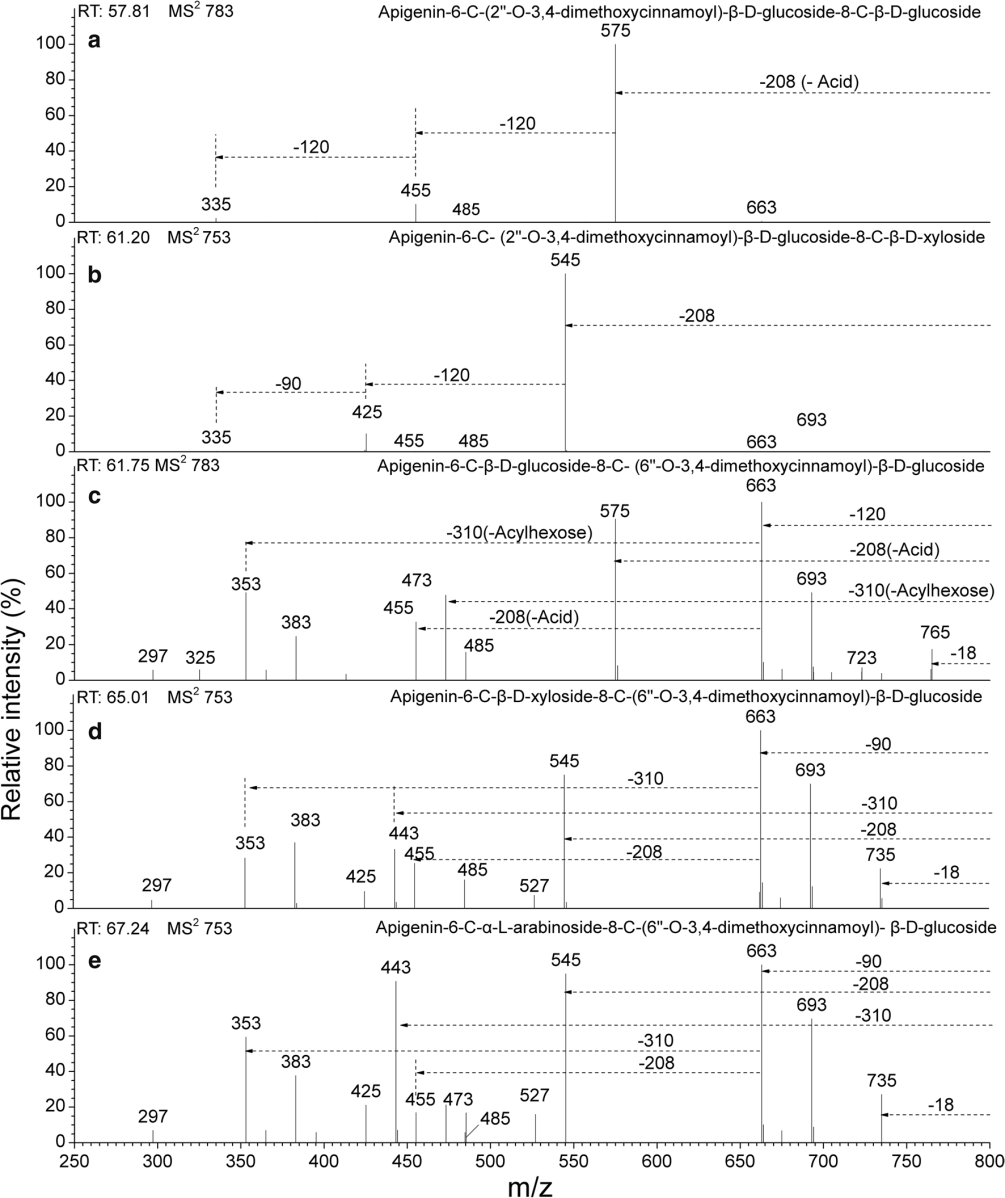
CID MS^2^ spectra fragmentations of five *C*-glycosyl flavones, acylated with 3,4-dimethoxycinnamoyl from the extracts of *Flickingeria fimbriata* in negative ionization mode: **a** Apigenin-6-*C*-(2″-*O*-3,4-dimethoxycinnamoyl)-β-d-glucoside-8-*C*-β-d-glucoside (19), **b** Apigenin-6-*C*-(2″-*O*-3,4-dimethoxycinnamoyl)-β-d-glucoside-8-*C*-β-d-xyloside (21), **c** Apigenin-6-*C*-β-d-glucoside-8-*C*-(6″-*O*-3,4-dimethoxycinnamoyl)-β-d-glucoside (22), **d** Apigenin-6-*C*-β-d-xyloside-8-*C*-(6″-*O*-3,4-dimethoxycinnamoyl)-β-d-glucoside (26), and **e** Apigenin-6-*C*-α-l-arabinoside-8-*C*-(6″-*O*-3,4-dimethoxycinnamoyl)-β-d-glucoside (29)

**Fig. 6:**
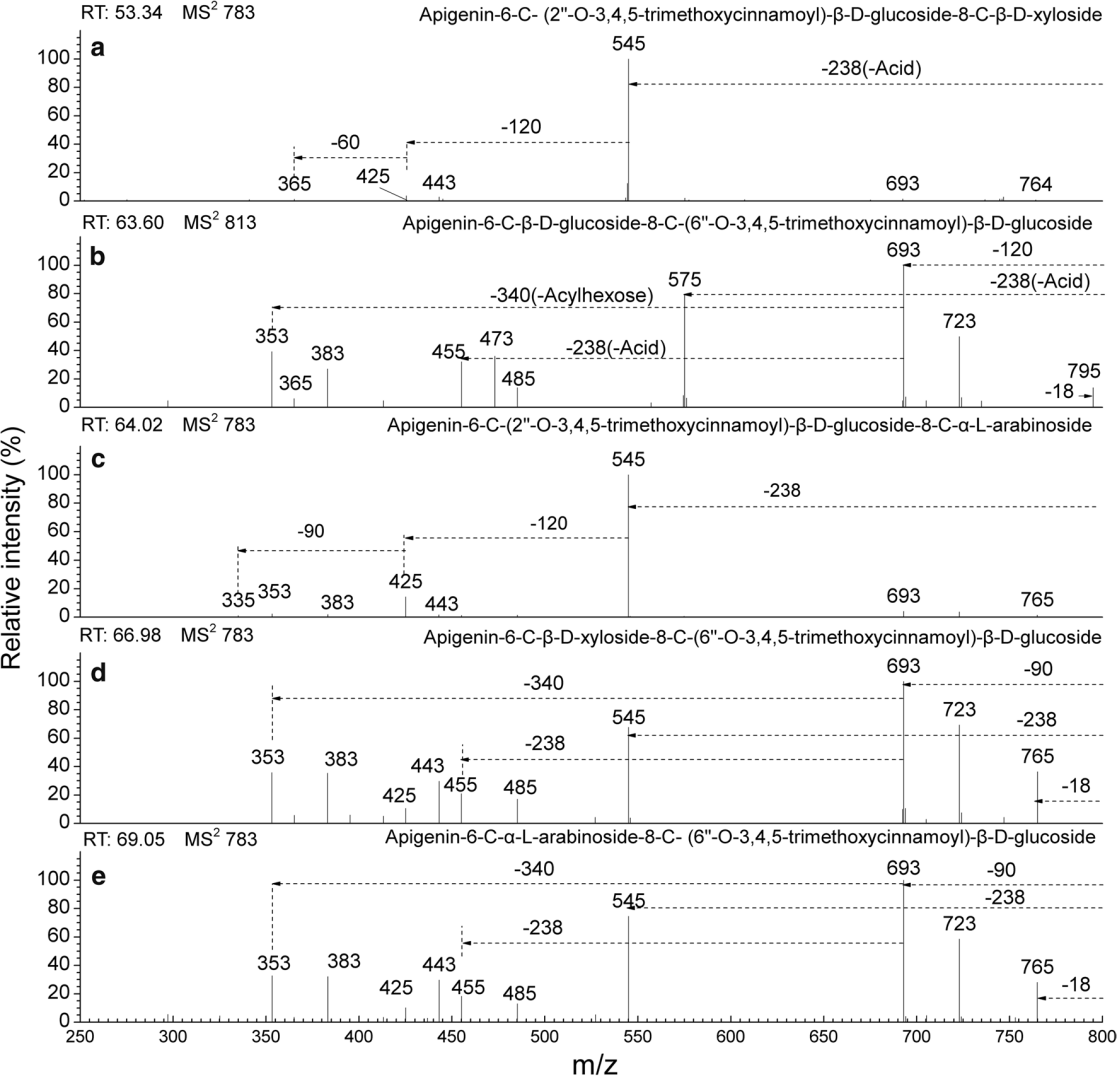
CID MS^2^ spectra fragmentations of five *C*-glycosyl flavones, acylated with 3,4,5-trimethoxycinnamoyl from the extracts of *Flickingeria fimbriata* in negative ionization mode: **a** Apigenin-6-*C*-(2″-*O*-3,4,5-trimethoxycinnamoyl)-β-d-glucoside-8-*C*-β-d-xyloside (15), **b** Apigenin-6-*C*-β-d-glucoside-8-*C*-(6″-*O*-3,4,5-trimethoxycinnamoyl)-β-d-glucoside (23), **c** Apigenin-6-*C*-(2″-*O*-3,4,5-trimethoxycinnamoyl)-β-d-glucoside-8-*C*-α-l-arabinoside (24), **d** Apigenin-6-*C*-β-d-xyloside-8-*C*-(6″-*O*-3,4,5-trimethoxycinnamoyl)-β-d-glucoside (28), and **e** Apigenin-6-*C*-α-l-arabinoside-8-*C*-(6″-*O*-3,4,5-trimethoxycinnamoyl)-β-d-glucoside (30)

The acyl group types were identified by neutral losses, which are characteristics of the acyl group or the acylated glycosyl residue. The acylation of *p*-coumaroyl and feruloyl in the hydroxyl of the *C*-glucosylation sugar showed higher polarity when compared to the acylation of 3,4-dimethoxycinnamoyl and 3,4,5-trimethoxycinnamoyl. These four types of acyl groups all belong to the derivatives of trans-cinnamoyl, but differ in the number of hydroxy and methoxy substituents. Characteristic acyl-related product ions [M-H-Acyl]^−^ and acid-related product ions [M-H-Acid]^−^ were observed in the former two types in the CID MS^2^ spectra, whereas in the latter two, only [M-H-Acid]^−^ was be detected. That is to say, the lower polarity acylated-*C*-glycosyl flavones are without the loss of the radical acyl group neutral fragments. In the CID MS^2^ spectra, the neutral fragment losses are 146 Da and 164 Da for *p*-coumaroyl (Fig. 3), and 176 Da and 194 Da for feruloyl (Fig. 4) in the hydroxyl of the *C*-glucosylation sugar, respectively [31], however, they are only 208 Da for 3,4-dimethoxycinnamoyl (Fig. 5) and only 238 Da for 3,4,5-trimethoxycinnamoyl (Fig. 6). Finally, the acylated *C*-glycosyl flavones we found are all acylated with *p*-coumaroyl, feruloyl, 3,4-dimethoxycinnamoyl or 3,4,5-trimethoxycinnamoyl on the hydroxyl in this work, and the majority of them are isomers.

The position of the acyl group in the hydroxyl of glucosyl flavones can also be identified by the characteristic of aglycone relative ions and the neutral fragments. When an acyl is in the 6″-*O* position on *C*-glucosylation glucose, simultaneous losses of acyl and of hexose residue, here called acylhexose, were obtained in fragment patterns, and the losses were 266 Da, 296 Da, 310 Da and 340 Da for *p*-coumaroyl hexose, feruloyl hexose, 3,4-dimethoxycinnamoylhexose, and 3,4,5-trimethoxycinnamoylhexose, respectively. To the best of our knowledge, this is the first report of 30 di-*C*-glycosyl flavones (Table 2) including ten non-acylated di-*C*-glycosyl flavones (Table 3), two acylated-Mono-*C*-glycosyl flavones (Table 4), twelve 6″-*O* -acylated-di-*C*-glucosyl flavones (Table 5) and six 2″-*O* -acylated-di-*C*-glucosyl flavones (Table 6) on systematic MS^n^ analysis in *F. fimbriata*.

**Table 2:** *C*-glycosyl flavones structures based on the aglycones of apigenin and chrysoeriol from *Flickingeria fimbriata*

	No.	R1	R2	R3	No.	R1	R2	R3
	1	Glu	Glu	H	16	Glu	Glu-b^2^	H
	2	Glu	Glu	OCH3	17	Glu-b^2^	Xyl	H
	3	Xyl	Glu	H	18	Ara	Glu-a^2^	H
	4	Glu	Ara	H	19	Glu-c^1^	Glu	H
	5	Glu	Xyl	H	20	Ara	Glu-b^2^	H
	6	Xyl	Glu	OCH3	21	Glu-c^1^	Xyl	H
	7	Glu	Ara	OCH3	22	Glu	Glu-c^2^	H
	8	Ara	Glu	OCH3	23	Glu	Glu-d^2^	H
	9	Glu	Xyl	H	24	Glu-d^1^	Ara	H
	10	Glu	Xyl	OCH3	25	Xyl	Glu-c^2^	OCH3
	11	H	Glu-a^1^	H	26	Xyl	Glu-c^2^	H
	12	Glu-a^1^	H	H	27	Xyl	Glu-d^2^	OCH3
	13	Glu-a^1^	Xyl	H	28	Xyl	Glu-d^2^	H
	14	Glu-b^1^	Xyl	H	29	Ara	Glu-c^2^	H
	15	Glu-d^1^	Xyl	H	30	Ara	Glu-d^2^	H

a: *ρ*-coumaroyl; b: feruloyl; c: 3,4-dimethoxycinnamoyl; d: 3,4,5-trimethoxycinnamoyl

^1^The position of acylation is set to 2″-*O* in sugar

^2^The position of acylation is set to 6″-*O* in sugar

**Table 3:** [M-H]^−^, MS^2^, MS^3^, and MS^4^ data of non-acylated di-*C*-glycosyl flavones in *Flickingeria fimbriata*

Peak No.	Rt (min)	Compounds	Molecular formula	[M-H]^−^ (*m/z*)	MS^2^ (*m/z*)^a^					MS^3^ (*m/z*)		MS^4^ (*m/z*)		
					− 120	− 90	− 60	[Ag-H+84]^−^	[Ag-H+114]^−^	[Ag-H+84]^−^	[Ag-H+114]^−^	[Ag-H+56]^−^	[Ag-H+28]^−^	[Ag-H+28-15]^−^
1	17.70	Vicenin-II	C_27_H_30_O_15_	593	473 (100)	503 (31)	–	353 (60)	383 (31)	353 (100)	383 (12)	325 (75)	297 (100)	–
3	20.71	Vicenin-I	C_26_H_28_O_14_	563	443 (61)	473 (100)	503 (60)	353 (83)	383 (56)	353 (100)	383 (9)	325 (70)	297 (100)	–
4	22.10	Schaftoside	C_26_H_28_O_14_	563	443 (100)	473 (65)	503 (5)	353 (74)	383 (45)	353 (100)	383 (20)	325 (81)	297 (100)	–
5	22.53	Isoschaftoside	C_26_H_28_O_14_	563	443 (84)	473 (100)	503 (69)	353 (93)	383 (97)	353 (100)	383 (19)	325(58)	297 (100)	–
9	25.30	Vicenin-III	C_26_H_28_O_14_	563	443 (100)	473 (61)	503 (3)	353 (46)	383 (32)	353 (100)	383 (13)	325 (97)	297 (100)	–
2	19.15	Chrys-6,8-di-*C*-β-d-glu	C_28_H_32_O_16_	623	503 (100)	533 (18)	–	383 (68)	413 (30)	383 (100)	413 (28)	355 (29)	327 (8)	312 (100)
6	22.70	Chrys-6-*C*-β-d-xyl-8-*C*-β-d-glu	C_27_H_30_O_15_	593	473 (100)	503 (41)	533 (26)	383 (87)	413 (50)	383 (100)	413 (44)	355 (51)	327 (2)	312 (100)
7	23.72	Chrys-6-*C*-β-d-glu-8-*C*-α-l-ara	C_27_H_30_O_15_	593	473 (100)	503 (36)	533 (28)	383 (71)	413 (55)	383 (100)	413 (46)	355 (32)	327 (4)	312 (100)
8	24.65	Chrys-6-*C*-α-l-ara-8-*C*-β-d-glu	C_27_H_30_O_15_	593	473 (90)	503 (100)	533 (5)	383 (85)	413 (36)	383 (93)	413 (100)	355 (15)	327 (7)	312 (100)
10^b^	26.79	Chrys-6-*C*-β-d-glu-8-*C*-β-d-xyl	C_27_H_30_O_15_	593	473 (77)	503 (74)	533 (8)	383 (100)	413 (47)	383 (100, MS^2^)	413 (45, MS^2^)	355 (3, MS^2^)	327 (4, MS^3^)	312 (100, MS^3^)

Main observed fragments. Other ions were found, but they have not been included

*Chrys* chrysoeriol, *glu* glucose, *ara* arabinoside, *xyl* xyloside

^a^Ag: aglycone, apigenin (Ag = 270); chrysoeriol (Ag = 300)

^b^MS^2^ and MS^3^ in bracket means the ions were obtained in MS^2^ and MS^3^, respectively

**Table 4:** [M-H]^−^, MS^2^, MS^3^, and MS^4^ data of acylated-mono-*C*-glycosyl flavone, when the position of acylation is set to 2″-*O* in the sugar in *Flickingeria fimbriata*

Peak No.	Rt (min)	Compounds	Molecular formula	[M-H]^−^	MS^2^ (*m/z*)^a^				MS^3^ (*m/z*)		MS^4^ (*m/z*)
					− 120	− 90	-Acid^b^	-Acid-120	[(Ag-H+42)-18]^−^	[(Ag-H+72)-18]^−^	[Ag-H+42-28]^−^
11	28.81	Apig-8-*C*-(2″-*O*-*ρ*-coum)-β-d-glu	C_30_H_26_O_12_	577	457 (7)	487 (0.6)	413 (100)	293 (26)	293 (100)	323 (0.7)	265 (49)
12	29.07	Apig-6-*C*-(2″-*O*-*ρ*-coum)-β-d-glu	C_30_H_26_O_12_	577	457 (8)	487 (0.9)	413 (100)	293 (60)	293 (100)	323 (1.4)	265 (62)

Main observed fragments. Other ions were found but they have not been included

Apig, Apigenin; glu, glucoside; *ρ*-coum, *ρ*-coumaroyl

^a^Ag: aglycone. apigenin (Ag = 270); chrysoeriol (Ag = 300)

^b^-Acid: *ρ*-coumaric acid (− 164 Da)

**Table 5:** [M-H]^−^,MS^2^, MS^3^, and MS^4^ data of acylated-di-*C*-glycosyl flavones, when the position of acylation is set to 6″-*O* in the sugar in *Flickingeria fimbriata*

Peak No.	Rt (min)	Compounds	Molecular formula	[M-H]^−^ (*m/z*)	MS^2^ (*m/z*)						MS^3^ (*m/z*)		MS^4^ (*m/z*)	
					− 120	− 90	− 60	-Acyl^a^	-Acid^b^	-Acylhexose^c^	[Ag-H+84]^−^	[Ag-H+114]^−^	[Ag-H+56]−	[Ag-H+28]^−^
17	54.13	Apig-6-*C*-(6″-*O*-fer)-β-d-glu-8-*C*-β-d-xyl	C_36_H_36_O_17_	739	618 (1)	649 (1.6)	679 (4)	563 (100)	545 (12)	443 (1)	353 (13)	383 (35)	–	–
16	53.83	Apig-6-*C*-β-d-glu-8-*C*-(6″-*O*-fer)-β-d-glu	C_37_H_38_O_18_	769	649 (100)	679 (56)	709 (5)	593 (17)	575 (35)	473 (32)	353 (100)	383 (14)	325 (86)	297 (100)
22	61.74	Apig-6-*C*-β-d-glu-8-*C*-(6″-*O*-3,4-dim)-β-d-glu	C_38_H_40_O_18_	783	663 (100)	–	723 (5)	–	575 (91)	473 (48)	353 (100)	383 (17)	325 (36)	297 (100)
23	63.60	Apig-6-*C*-β-d-glu-8-*C*-(6″-*O*-3,4,5-trim)-β-d-glu	C_39_H_42_O_19_	813	693 (100)	723 (50)	753 (1)	–	575 (79)	473 (36)	353 (100)	383 (11)	–	–
18	56.89	Apig-6-*C*-α-l-ara-8-*C*-(6″-*O*-*ρ*-coum)β-d-glu	C_35_H_34_O_16_	709	–	619 (100)	649 (54)	563 (9)	545 (12)	443 (11)	353 (100)	383 (36)	325 (30)	297 (100)
20	57.93	Apig-6-*C*-α-l-ara-8-*C*-(6″-*O*-fer)-β-d-glu	C_36_H_36_O_17_	739	–	649 (100)	679 (69)	563 (11)	545 (1.5)	443 (19)	353 (100)	383 (16)	325 (59)	297 (100)
26	65.01	Apig-6-*C*-β-d-xyl-8-*C*-(6″-*O*-3,4-dim)-β-d-glu	C_37_H_38_O_17_	753	–	663 (100)	693 (70)	–	545 (75)	443 (33)	353 (100)	383 (16)	325 (58)	297 (100)
28	66.97	Apig-6-*C*-β-d-xyl-8-*C*-(6″-*O*-3,4,5-trim)-β-d-glu	C_38_H_40_O_18_	783	–	693 (100)	723 (69)	–	545 (68)	443 (30)	353 (67)	383 (2)	325 (2)	–
29	67.24	Apig-6-*C*-α-l-ara-8-*C*-(6″-*O*-3,4-dim)-β-d-glu	C_37_H_38_O_17_	753	–	663 (100)	693 (70)	–	545 (95)	443 (91)	353 (100)	383 (9)	325 (100)	297 (92)
30	69.04	Apig-6-*C*-α-l-ara-8-*C*-(6″-*O*-3,4,5-trim)-β-d-glu	C_38_H_40_O_18_	783	–	693 (100)	723 (58)	–	545 (74)	443 (30)	353 (100)	383 (32)	325 (99)	297 (100)
25	64.79	Chrys-6-*C*-β-d-xyl-8-*C*-(6″-*O*-3,4-dim)-β-d-glu	C_38_H_40_O_18_	783	–	693 (79)	723 (49)	–	575 (72)	473 (100)	383 (100)	413 (44)	355 (37)	327 (8)
27	66.52	Chrys-6-*C*-β-d-xyl-8-*C*-(6″-*O*-3,4,5-trim)-β-d-glu	C_38_H_40_O_18_	813	–	723 (76)	753 (55)	–	575 (71)	473 (100)	383 (100)	413 (36)	355 (33)	327 (2.4)

Main observed fragments. Other ions were found, but they have not been included

Apig, Apigenin; Chrys, Chrysoeriol; glu, glucoside; ara, arabinoside; xyl, xyloside; *ρ*-coum, *ρ*-coumaroyl; fer, feruloyl; 3,4-dim, 3,4-dimethoxycinnamoyl; 3,4,5-trim, 3,4,5-trimethoxycinnamoyl

^a^-Acyl: *ρ*-coumaroyl (− 146 Da); feruloyl (− 176 Da)

^b^-Acid: *ρ*-coumaric acid (− 164 Da); ferulic acid (− 194 Da); 3,4-dimethoxycinnamoyl acid (− 208 Da); 3,4,5-trimethoxycinnamoyl acid (− 238 Da)

^c^-Acylhexose: *ρ*-coumaroylhexose (− 266 Da); feruloylhexose (− 296 Da); 3,4-dimethoxycinnamoylhexose (− 310 Da); 3,4,5-trimethoxycinnamoylhexose (− 340 Da)

**Table 6:** [M-H]^−^,MS^2^, MS^3^, and MS^4^ data of acylated-di-*C*-glucosyl flavones, when the position of acylation is set to 2″-*O* in the sugar in *Flickingeria fimbriata*

Peak No.	Rt (min)	Compounds	Molecular formula	[M-H]^−^ (*m/z*)	MS^2^ (*m/z*)						MS^3^ (*m/z*)	MS^4^ (*m/z*)	
					− 120	− 90	− 60	-Acyl^a^	-Acid^b^	-Acid-120	− 120	[(Ag-H+84)-18]^−^	[(Ag-H+114)-18]^−^
13	51.64	Apig-6-*C*-(2″-*O*-*ρ*-coum)-β-d-glu-8-*C*-β-d-xyl	C_35_H_34_O_16_	709	–	619 (1.5)	649 (0.5)	563 (18)	545 (100)	425 (6)	425 (100)	335 (100)	365 (49)
14	53.07	Apig-6-*C*-(2″-*O*-fer)-β-d-glu-8-*C*-β-d-xyl	C_36_H_36_O_17_	739	–	649 (0.3)	679 (0.4)	563 (9)	545 (100)	425 (9)	425 (100)	335 (100)	365 (50)
19	57.81	Apig-6-*C*-(2″-*O*-3,4-dim)-β-d-glu-8-*C*-β-d-glu	C_38_H_40_O_18_	783	663(0.7)	–	–	–	575 (100)	455 (10)	455 (100)	335 (100)	365 (3)
21	61.19	Apig-6-*C*-(2″-*O*-3,4-dim)-β-d-glu-8-*C*-β-d-xyl	C_37_H_38_O_17_	753	–	663 (0.1)	693(0.1)	–	545 (100)	425 (10)	425 (100)	335 (100)	365 (45)
15	53.33	Apig-6-*C*-(2″-*O*-3,4,5-trim)-β-d-glu-8-*C*-β-d-xyl	C_38_H_40_O_18_	783	–	693 (1.5)	–	–	545 (100)	425 (4)	425 (100)	335 (100)	365 (79)
24	64.02	Apig-6-*C*-(2″-*O*-3,4,5-trim)-β-d-glu-8-*C*-α-l-ara	C_38_H_40_O_18_	783	663(0.2)	693 (4)	723(4)	–	545 (100)	425 (14)	425 (100)	335 (100)	365 (47)

Main observed fragments. Other ions were found but they have not been included

Apig, Apigenin; glu, glucoside; ara, arabinoside; xyl, xyloside; *ρ*-coum, *ρ*-coumaroyl; fer, feruloyl; 3,4-dim, 3,4-dimethoxycinnamoyl; 3,4,5-trim, 3,4,5-trimethoxycinnamoyl

^a^-Acyl: *ρ*-coumaroyl (− 146 Da); feruloyl (− 176 Da)

^b^-Acid: *ρ*-coumaric acid (− 164 Da); ferulic acid (− 194 Da); 3,4-dimethoxycinnamoyl acid (− 208 Da); 3,4,5-trimethoxycinnamoyl acid (− 238 Da)

### HPLC–ESI–MS^n^ analysis of non-acylated-di-*C*-glycosyl flavones

For compounds **1** and **2**, the base peak of fragment ions [(M-H)-120]^−^ in the CID MS^2^ spectra and the base peak ions [(M-H-120)-120]^−^ in the MS^3^ spectra suggested that the simultaneous glucosylation of glucose occurred in positions 6 and 8 [32]. A pair of fragments at *m/z* 353 [Ag(270)-H+84]^−^ and 383 [Ag(270)-H+114]^−^ in the MS^3^ spectra indicated that the nucleus of flavonoids is apigenin [33]. The coinstantaneous presence of fragments at *m/z* 383 for [Ag(300)-H+84]^−^ and 411 for [Ag(300)-H+114]^−^ in the MS^3^ spectra indicated that the nucleus of flavonoids is chrysoeriol [29]. In both nucleuses of the flavones above, the presence of [Ag-H+56]^−^ and [Ag-H+28]^−^ were easily detected by the loss of 28 Da (CO) and 56 Da (2CO) at the position of 6-*C* or/and 8-*C*, respectively. However, [Ag-H+28-15]^−^ could only be detected in the fragments of chrysoeriol-di-*C*-glycosyl flavone by the loss of 15 Da (CH_3_) [5]. From all the results above, the compounds were found to be apigenin-6,8-di-*C*-β-d-glucoside (**1**, vicenin-II) and chrysoeriol-6,8-di-*C*-β-d-glucoside (**2**). In the compounds **3**, **5**, **6**, and **8**, di-*C*-glycosyl flavone was also typical due to [Ag-H+84]^−^ and [Ag-H+114]^−^. Base peak ions [(M-H)-90]^−^ and ions [(M-H)-60]^−^ in MS^2^ as well as base peak ions [(M-H-90)-120]^−^ ([Ag-H+84]^−^) in MS^3^ indicated the structure of Ag-6-*C*-pentose-8-*C*-hexose. In the compounds **4**, **7**, **9**, and **10**. Additionally, the fragment ions [(M-H-90)-90]^−^ and [(M-H-60)-120]^−^ in MS^2^ from the 8-*C*-arabinoside flavone were produced more extensively than 8-*C*-xyloside. The compounds were found to be apigenin-6-*C*-β-d-xyloside-8-*C*-β-d-glucoside (**3**, vicenin-I) and apigenin-6-*C*-α-l-arabinoside-8-*C*-β-d-glucoside (**5**, isoschaftoside), chrysoeriol-6-*C*-β-d-xyloside-8-*C*-β-d-glucoside (**6**), chrysoeriol-6-*C*-α-l-arabinosidee-8-*C*-β-d-glucoside (**8**). In compounds **4**, **7**, **9**, and **10**, the base peak ions [(M-H)-120]^−^ and ions [(M-H)-90]^−^ in MS^2^, and base peak ions [(M-H-120)-90]^−^ ([Ag-H+84]^−^), apparent in MS^3^, indicated the structure of Ag-6-*C*-hexose-8-*C*-pentose. Fragment ions [(M-H-120)-90]^−^ and [(M-H-90)-90]^−^ in the MS^2^ spectra were produced more extensively for the 8-*C*-arabinoside flavone as opposed to 8-*C*-xyloside. Considering the results obtained and the reported identification [33], we describe these compounds as apigenin-6-*C*-β-d-glucoside-8-*C*-α-l-arabinoside (**4**, schaftoside), apigenin-6-*C*-β-d-glucoside-8-*C*-β-d-xyloside (**9**, vicenin-III), chrysoeriol-6-*C*-β-d-glucoside-8-*C*-α-l-arabinoside (**7**), and chrysoeriol-6-*C*-β-d-glucoside-8-*C*-β-d-xyloside (**10**) (Table 3). Compounds **1**, **3**, **4**, **5**, and **9** were confirmed by the reference standards using the eluted order and retention time.

### HPLC–ESI–MS^n^ analysis of acylated-*C*-glycosyl flavones

For compounds **11** and **12**, due to the higher relative intensity produced by ions at *m/z* 293 [(M-H-164)-120]^−^ in MS^2^ in compound **12** relative to compound **11**, together with the base peak ions [(Ag-H+42)-18]^−^ obtained in the MS^3^ spectra, the characteristic of an acylation in the 2″-*O* position on mono-*C*-glycosyl flavones was confirmed (Table 4, Fig. 3a, b). 6-*C*-2″-*O*-*c*oumaroyl-isomers can eliminate the acid between the 2″-*O*-coumaroyl group of sugars and the 5- and 7-hydroxyl groups of aglycone, but 8-*C*-2″-*O*-coumaroyl-isomers only respond to the 7-hydroxyl group [27], which results in 6-*C*-2″-*O*-coumaroyl isomers producing more acid-related ions. 8-*C*-glycoside compounds, eluted before their 6-*C* isomer, also help distinguish the isomers [8]. Consequently, the compounds were deduced to be apigenin-8-*C*-(2″-*O*-coumaroyl)-β-d-glucoside (**11**) and apigenin-6-*C*-(2″-*O*-coumaroyl)-β-d-glucoside (**12**).

For compounds **16**, **18**, and **20**, base peak ions [M-H-120]^−^ or [M-H-90]^−^ and ions [M-H-266/296]^−^ in the MS^2^ spectra showed the characteristic of a 6-*C*-pentose/henose-8-*C*-(6″-*O*-*p*-coumaroyl/feruloyl)-henose flavone (Figs. 3d, 4b, d**)**. Ions [Ag-H+84]^−^ and [Ag-H+114]^−^ were obtained in the MS^3^ spectra, which further confirmed the acylation at the 6″-*O* position (Table 5). The compound was determined to be apigenin-6-*C*-β-d-glucoside-8-*C*-(6″-*O*-feruloyl)-β-d-glucoside (**16**). Ions [(M-H-90)-Acylhexose]-(or [Ag-H+84]^−^), found to be produced at the base peak in MS^3^, prove the glucosylation of arabinose. It is likely that the compounds were apigenin-6-*C*-α-l-arabinoside-8-*C*-(6″-*O*-coumaroyl)β-d-glucoside (**18**) and apigenin-6-*C*-α-l-arabinoside-8-*C*-(6″-*O*-feruloyl)-β-d-glucoside (**20**).

For compounds **22**, **23**, **25**–**30**, besides the aglycone ions [Ag-H+84]^−^ and [Ag-H+114]^−^ in the MS^4^ spectra (Table 5), base peak ions [M-H-120]^−^ together with ions [(M-H-120)-310/340]^−^ or [M-H-90]^−^ with [(M-H-90)-310/340]^−^ in the MS^2^ spectra (Figs. 5c–e and 6b, d, e**)** characterize 6-*C*-pentose/henose-8-*C*-(6″-*O*-3, 4-dimethoxycinnamoyl/3,4,5-trimethoxycinnamoyl)-henose flavones. The compounds were determined to be apigenin-6-*C*-β-d-glucoside-8-*C*-(6″-*O*-3,4-dimethoxycinnamoyl)-β-d-glucoside (**22**) and apigenin-6-*C*-β-d-glucoside-8-*C*-(6″-*O*-3,4,5-trimethoxycinnamoyl)-β-d-glucoside (**23**). For compounds **26** and **29**, when arabinose was substituted at 6-*C*, 353[(M-H-90)-310]^−^ was abundant in the MS^2^ spectra (Fig. 5d, e), whereas these ions showed a low relative intensity in MS^2^ for xylose substitution. As well, for compounds **28** and **30**, 353[(M-H-90)-340]^−^ even became the base peak in the MS^3^ spectra for compounds **30**, whereas these ions showed a lower relative intensity in MS^3^ for compounds **28** (Table 5). From the results above, the compounds were determined to be chrysoeriol-6-*C*-β-d-xyloside-8-*C*-(6″-*O*-3,4-dimethoxycinnamoyl)-β-d-glucoside (**25**), apigenin-6-*C*-β-d-xyloside-8-*C*-(6″-*O*-3,4-dimethoxycinnamoyl)-β-d-glucoside (**26**), chrysoeriol-6-*C*-β-d-xyloside-8-*C*-(6″-*O*-3,4,5-trimethoxycinnamoyl)-β-d-glucoside (**27**), apigenin-6-*C*-β-d-xyloside-8-*C*-(6″-*O*-3,4,5-trimethoxycinnamoyl)-β-d-glucoside (**28**), apigenin-6-*C*-α-l-arabinoside-8-*C*-(6″-*O*-3,4-dimethoxycinnamoyl)-β-d-glucoside (**29**), and apigenin-6-*C*-α-l-arabinoside-8-*C*-(6″-*O*-3,4,5-trimethoxycinnamoyl)-β-d-glucoside (**30**).

For compounds **13**, **14**, **15**, **19**, **21**, and **24**, the base peak fragment [(M-H)-Acid]^−^ without a loss of the neutral fragments of acylhexose, as well as aglycone-related ions [(Ag-H+84)-18]^−^ and [(Ag-H+114)-18]^−^ in MS^4^ demonstrated the characteristics of 6-*C*-(2″-*O*-*p*-coumaroyl/feruloyl/3,4-dimethoxycinnamoyl/3,4,5-trimethoxycinnamoyl)-henose-8-*C*-pentose/henose flavones (Table 6). In terms of the base peak ions [(M-H-208)-120]^−^ in the MS^3^ spectra and ions [(M-H-208)-120-120]^−^ (or [(Ag-H+84)-18]^−^), found in MS^4^, the compound was determined to be apigenin-6-*C*-[2″-*O*-(3,4-dimethoxycinnamoyl)]-β-d-glucoside-8-*C*-β-d-glucosides (**19**) (Table 6). In compounds **13** and **14**, by considering the characteristics of the substitutions of xyloside, a low relative intensity of ions [M-H-60]^−^ and [M-H-90]^−^ in the MS^2^ spectra. The compounds were tentatively assigned as apigenin-6-*C*-(2″-*O*-*p*-coumaroyl)-β-d-glucoside-8-*C*-β-d-xyloside (**13**) and apigenin-6-*C*-(2″-*O*-feruloyl)-β-d-glucoside-8-*C*-β-d-xyloside (**14**) (Figs. 3c and 4a). In compounds **21**, **15** and **24**, besides the base peak ions 335[(M-H-208/238-120)-90]^−^ (or [(Ag-H+84)-18]^−^) in MS^4^ (Table 6), compared to the xylose substitution glucosylation flavones, the arabinose substitution glycosyl flavones had a greater abundance of ions 425[(M-H-208/238)-120]^−^ in MS^2^ (Fig. 6a, c). The compounds were determined to be apigenin-6-*C*-(2″-*O*-3,4,5-trimethoxycinnamoyl)-β-d-glucoside-8-*C*-β-d-xyloside (**15**), apigenin-6-*C*-(2″-*O*-3,4-dimethoxycinnamoyl)-β-d-glucoside-8-*C*-β-d-xyloside (**21**), and apigenin-6-*C*-(2″-*O*-3,4,5-trimethoxycinnamoyl)-β-d-glucoside-8-*C*-α-l-arabinoside (**24**).

For compound **17**, the base peak ion [(M-H)-Acyl]^−^ in the MS^2^ spectra, and the characteristic ions [Ag-H+114]^−^, [Ag-H+84]^−^ in MS^4^, are characteristic of 6-*C*-(6″-*O*-*p*-coumaroyl/feruloyl)-henose-8-*C*-pentose/henose flavones (Table 5). According to the low relative intensity of [(M-H-176)-120]^−^ in the MS^2^ spectra (Fig. 4), the compounds were supposed to be apigenin-6-*C*-(6″-*O*-feruloyl)-β-d-glucoside-8-*C*-β-d-xyloside (**17**).

### Method validation

The standard curve regressions were based on data from five concentrations of each standard solution. The peak areas and standard concentrations of each flavonoid compound were linearly fitted to a linear relation of Y = AX + B, where X represents the injection amount (μg) and Y represents the peak area, measured by HPLC. The correlation coefficients were also calculated. As listed in Table 7, all the calibration curves showed good linearity in the injection amount range (μg) (*R*^*2*^ > 0.999). The precision RSDs of the 5 compounds were 0.84–1.97%. The values for repeatability were 0.75–2.19%. To confirm the stability, a standard solution mixed with methanol was analyzed at 0, 2, 4, 8, 12, and 24 h to evaluate the stability of the solution. The results showed that the stability RSD ranged from 1.11 to 2.12%. The results showed that the HPLC method for vicenin-II, vicenin-I, schaftoside, isoschaftoside, and vicenin-III had an average assay recovery between 100.55 and 102.68% and a good reproducibility RSD ranged 0.68–1.49%. All the data indicated that this method is satisfactory for the qualitative and quantitative analysis of *F. fimbriata*.

**Table 7:** Linear regression and precision data of vicenin-II, vicenin-I, schaftoside, isoschaftoside, and vicenin-III

Compound	Linear regression calibration curves	R^2^	Injection amount range (μg)	Repeatability RSD (%)	Precision RSD (%)	Stability RSD (%) (n **= **6)	Reproducibility (n = 6)	
							Mean	RSD (%)
Vicenin-II	Y = 1811.9 X + 15.284	0.9998	0.030–1.782	0.75	0.84	1.11	100.55	0.68
Vicenin- I	Y = 2324.2X + 38.975	0.9998	0.029–3.234	1.11	1.65	2.12	102.68	1.49
Schaftoside	Y = 2373.1 X + 17.333	0.9996	0.028–1.687	1.45	1.89	1.55	101.42	1.24
Isoschaftoside	Y = 2514.8 X + 16.737	0.9997	0.028–1.687	0.88	1.24	1.46	101.39	1.05
Vicenin-III	Y = 2282.5 X − 19.708	0.9999	0.030–3.288	2.19	1.97	1.87	100.86	1.21

### Sample quantitative analysis

**Fig. 7:**
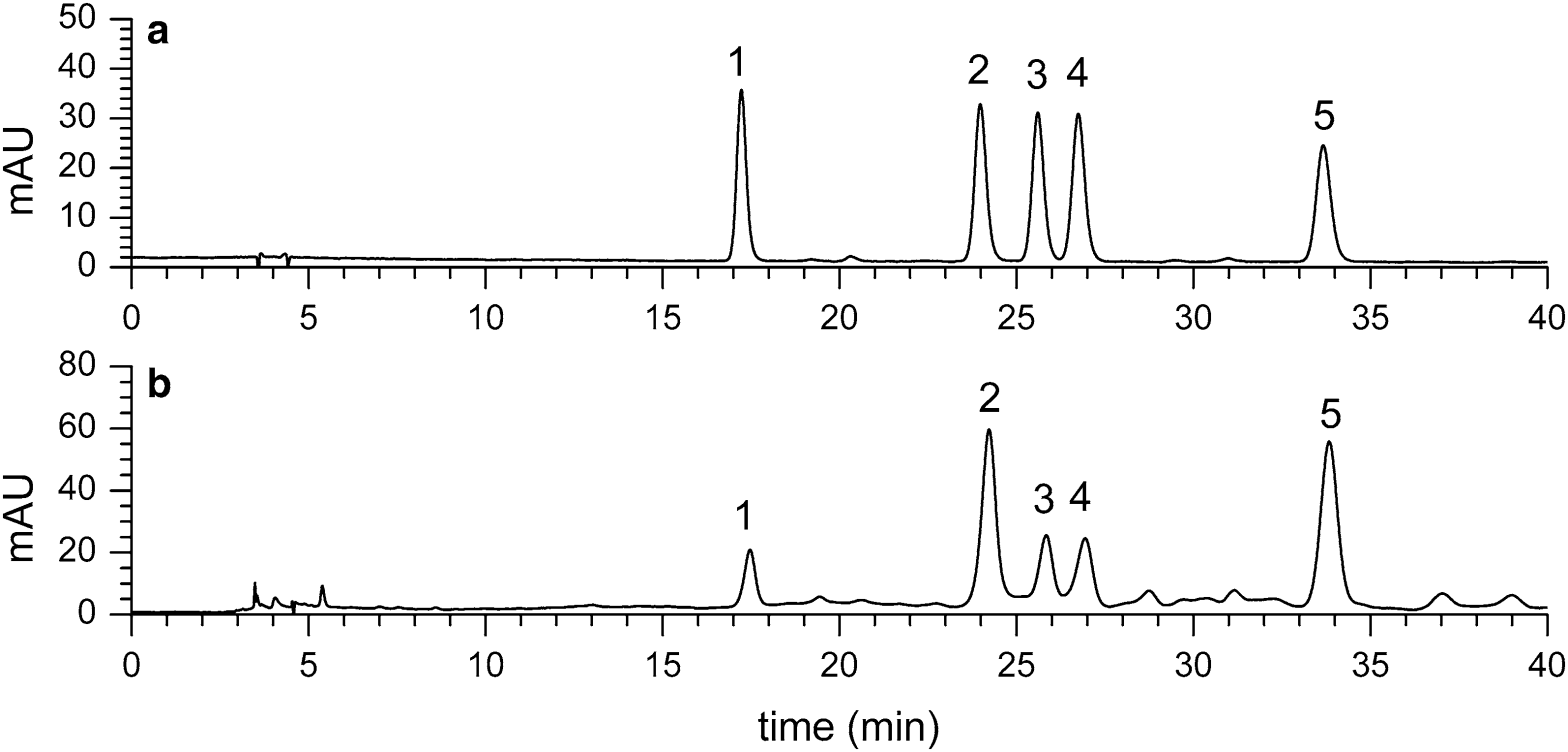
Qualitative analysis in the extracts of *Flickingeria fimbriata*: **a** The HPLC chromatogram of the five standards (1. vicenin-II; 2. vicenin-I; 3. schaftoside; 4. isoschaftoside; 5. vicenin-III); **b** the representative HPLC chromatogram of *Flickingeria fimbriata* (FF4)

**Fig. 8:**
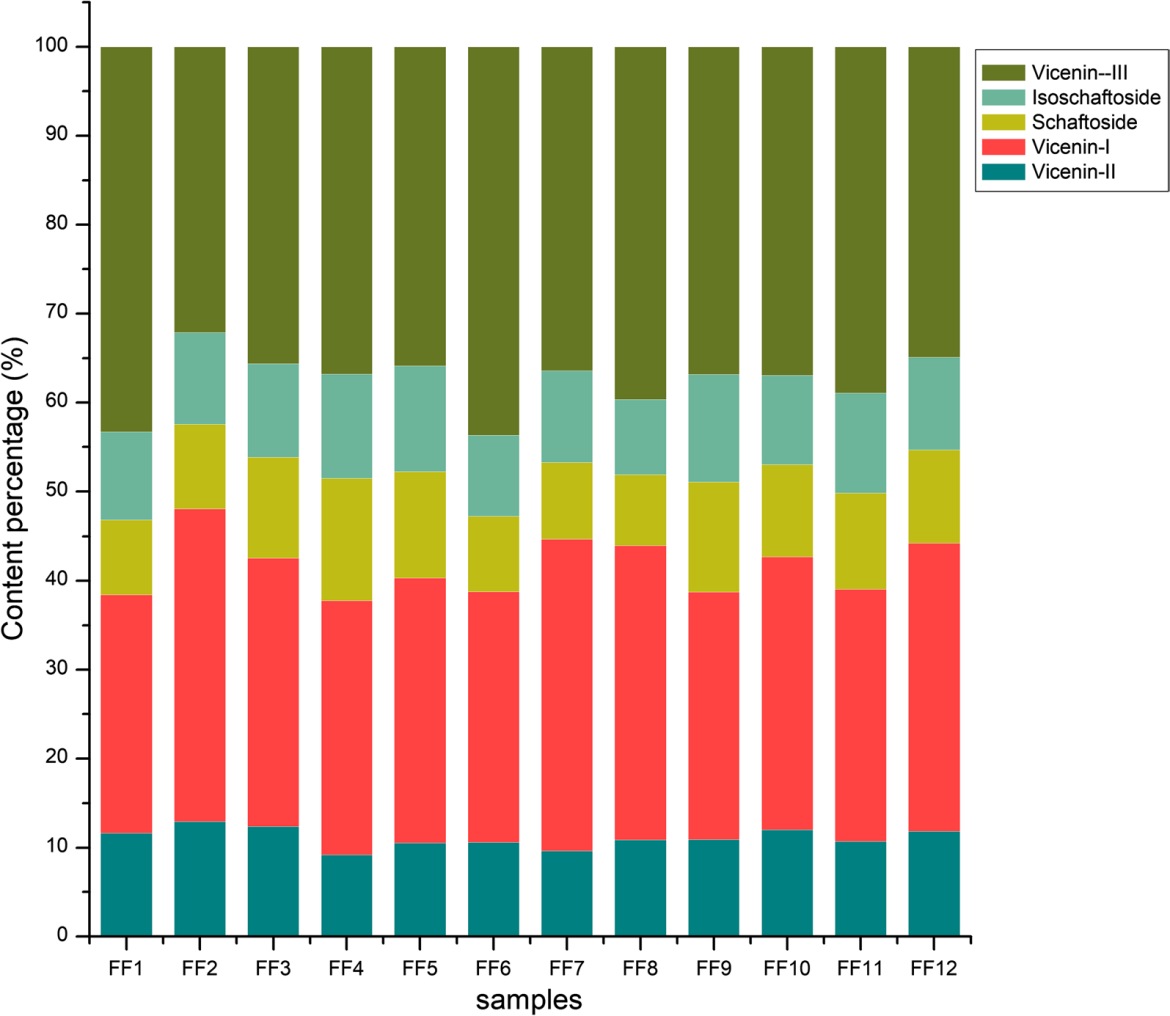
Content percentage of five main *C*-glycosyl flavones in *Flickingeria fimbriata*, including vicenin-II, vicenin-I, schaftoside, isoschaftoside, and vicenin-III

The proposed HPLC method was applied to analyze the five main compounds in the 12 batches of *F. fimbriata* samples from Guangdong, Yunnan, and Sichuan provinces in South China. The chromatogram of the five standards and the representative chromatogram of *F. fimbriata* (sample FF4) used for quantification were shown in Fig. 7. The contents of vicenin-I and vicenin-III in all 12 batches of samples were higher than the other three compounds, as shown in Fig. 8. These quantitative results were in accordance with the results from MS^n^ analysis, which showed that there was a greater acyl group substitution in vicenin-I and vicenin-III as well as pentose substitution on the flavonoid, referred to as xylose, as opposed to arabinose. Vicenin-II, vicenin-I, schaftoside, isoschaftoside, and vicenin-III were all found to be higher in the samples FF2, FF3, FF4, FF6, and FF10, as shown in Table 8. The differences in contents are due to many factors, including the growth environment, harvesting time, and growing years. Due to the lack of samples from different origins, this phenomenon should be studied in the future.

**Table 8:** The contents of vicenin-II, vicenin-I, schaftoside, isoschaftoside, and vicenin-III in *Flickingeria fimbriata*

Samples	Dried weight (mg/g)*				
	Vicenin-II (1)	Vicenin-I (3)	Schaftoside (4)	Isoschaftoside (5)	Vicenin-III (9)
FF1	0.227 ± 0.003	0.525 ± 0.005	0.164 ± 0.005	0.194 ± 0.005	0.847 ± 0.018
FF2	0.742 ± 0.023	2.019 ± 0.049	0.544 ± 0.021	0.593 ± 0.021	1.846 ± 0.017
FF3	0.748 ± 0.002	1.822 ± 0.017	0.683 ± 0.005	0.635 ± 0.008	2.154 ± 0.014
FF4	0.331 ± 0.014	1.031 ± 0.045	0.496 ± 0.021	0.421 ± 0.019	1.328 ± 0.042
FF5	0.162 ± 0.005	0.588 ± 0.009	0.145 ± 0.009	0.173 ± 0.015	0.611 ± 0.048
FF6	0.480 ± 0.014	1.462 ± 0.022	0.351 ± 0.010	0.373 ± 0.008	1.752 ± 0.014
FF7	0.201 ± 0.005	0.570 ± 0.015	0.228 ± 0.017	0.227 ± 0.010	0.687 ± 0.012
FF8	0.137 ± 0.002	0.350 ± 0.008	0.155 ± 0.003	0.152 ± 0.007	0.463 ± 0.004
FF9	0.196 ± 0.009	0.519 ± 0.017	0.197 ± 0.010	0.206 ± 0.015	0.712 ± 0.039
FF10	0.518 ± 0.015	1.419 ± 0.035	0.457 ± 0.010	0.456 ± 0.012	1.527 ± 0.029
FF11	0.145 ± 0.006	0.388 ± 0.008	0.116 ± 0.008	0.125 ± 0.010	0.600 ± 0.024
FF12	0.232 ± 0.006	0.594 ± 0.004	0.120 ± 0.008	0.194 ± 0.009	0.715 ± 0.045

*The values are expressed as mean ± standard deviation, *n *= 2

### Contents of five di-*C*-glycosyl flavones in *Flickingeria fimbriata*

Quantification was based on an external standard method using calibration curves fitted by linear regression analysis. The validated HPLC method was subsequently applied to the determination of 12 batches of *F. fimbriata*, and the quantitative analysis of the five main di-*C*-glycosyl flavones are summarized in Table 8. The quality of the *F. fimbriata* extracts was assessed by determining their flavonoid content. The contents of the 5 main di-*C*-glycosyl flavones eluted in the order of vicenin-II, vicenin-I, schaftoside, isoschaftoside, then vicenin-III, and the dried herbal material contents of them were 0.137–0.748 mg/g, 0.388–2.019 mg/g, 0.116–0.683 mg/g, 0.125–0.635 mg/g, and 0.463–2.154 mg/g, respectively.

## Conclusion

*Flickingeria fimbriata* benefited by its superiority of medicine food homology which is widely applied in health industry. However, only morphological identification and microscopic identification methods reported in Guangdong Chinese Materia Medicine Standards indicated a lack of quality control. In this study, 20 acylated *C*-glycosyl flavones and 10 non-acylated *C*-glycosyl flavones were characterized for the first time in *F. fimbriata* using HPLC-DAD and HPLC–ESI–MS^n^, which laid a foundation of improving the standard for quality control and identification of *F. fimbriata*.

The methods allowed us to identify several important structural characteristics of *C*-glycosyl flavones including (1) the nature of aglycone (apigenin or chrysoeriol) (2) types of sugar units (glucose, arabinose, or xyloside), (3) glycosylation position (6-*C* or 8-*C*), (4) types of acyl groups (*p*-coumaroyl, feruloyl, 3,4-dimethoxycinnamoyl, or 3,4,5-trimethoxycinnamoyl), and (5) acylation position (2″-*O* or 6″-*O*). Additionally, we found the isomers of 6-*C* and 8-*C*-glycosyl flavones almost coexisted in *F. fimbriata*. The acylated flavones also had following characteristics in this plants, when acylation occurs at the 2″-*O* position, sugar substitution for 6-*C*-glucosylation is more likely. When acylation occurred at the 6″-*O* position, sugar substitution for 8-*C*-glucosylation is more likely. And the acyl groups in the compounds analyzed in this article were all substituted on glucose.

Although we lacked samples from the same growth environment, with the same harvesting time or same growing years, the contents of vicenin-I and vicenin-III were found to be higher than those of vicenin-II, schaftoside, and isoschaftoside in the quantitative analysis of all 12 batches of samples, and vicenin-I and vicenin-III were revealed to be more strongly acylated based on the results, which are considered to be a characterization of flavonoids in *F. fimbriata*.

## Data Availability

The data and materials used and/or analyzed during the current study are available from the corresponding author on reasonable request.

## Abbreviations

TCM: traditional Chinese medicine
HPLC: high-performance liquid chromatography
HPLC–ESI–MS^n^: high performance liquid chromatography–electrospray ionization–multiple stage tandem mass spectrometry
DAD: diode array detector
MSn: multiple stage tandem mass spectrometry
IT-MS: ion-trap mass spectrometer
FF: *Flickingeria fimbriata*
RT: retention times

## References

[1] Li H, Zhao J, Chen J, Zhu L, Wang D, Jiang L (2015). Diterpenoids from aerial parts of Flickingeria fimbriata and their nuclear factor-kappaB inhibitory activities. Phytochemistry.

[2] Ding G, Fei J, Wang J, Xie Y, Li R, Gong N (2016). Fimbriatols A-J, highly oxidized ent-kaurane diterpenoids from traditional chinese plant *Flickingeria fimbriata* (B1) Hawkes. Sci Rep.

[3] Chen JL, Zhong WJ, Tang GH, Li J, Zhao ZM, Yang DP (2014). Norditerpenoids from *Flickingeria fimbriata* and their inhibitory activities on nitric oxide and tumor necrosis factor-α production in mouse macrophages. Molecules.

[4] Wu YP, Liu WJ, Zhong WJ, Chen YJ, Chen DN, He F (2017). Phenolic compounds from the stems of *Flickingeria fimbriata*. Nat Prod Res.

[5] Tezuka Y, Yoshida Y, Kikuchi T, Xu G (1993). Constituents of *Ephemerantha fimbriata*. Isolation and structure elucidation of two new phenanthrenes, fimbriol-A and fimbriol-B, and a new dihydrophenanthrene, ephemeranthol-C. Chem Pharm Bull.

[6] Ali A, Abdullah S, Hammid H, Ali M, Alam M (2003). A new sterol from the pseudobulb of Desmotrichum fimbriatum Blume. Pharmazie.

[7] Huang YC, Ren J, Zhu S, Xie ZS, Ye JH, Wei G (2015). Analysis of HPLC characteristic spectrum of Hera Flickingeriae. Trad Chinese Drug Res Clin Pharmacol.

[8] Becchi M, Fraisse D (1989). Fast atom bombardment and fast atom bombardment collision-activated dissociation mass-analyzed ion kinetic-energy analysis of C-glycosidic flavonoids. Biomed Environ Mass Spectrom.

[9] Kachlicki P, Piasecka A, Stobiecki M, Marczak L (2016). Structural characterization of flavonoid glycoconjugates and their derivatives with mass spectrometric techniques. Molecules.

[10] Zhou CH, Xie ZS, Lei ZX, Huang YC, Wei G (2018). Simultaneous identification and determination of flavonoids in *Dendrobium officinale*. Chem Cent J.

[11] Zhang H, Zhang D, Ray K, Zhu M (2009). Mass defect filter technique and its applications to drug metabolite identification by high-resolution mass spectrometry. J Mass Spectrom.

[12] Kargutkar S, Brijesh S (2017). Anti-inflammatory evaluation and characterization of leaf extract of *Ananas comosus*. Inflammopharmacology.

[13] Zanyatkin I, Stroylova Y, Tishina S, Stroylov V, Melnikova A, Haertle T (2017). Inhibition of prion propagation by 3,4-dimethoxycinnamic acid. Phytother Res.

[14] Ding G, Wang J, Fei JD, Li RT, Jia HM, Zhang T (2017). Fimbrialtols K-M, highly functionalized ent -kaurane diterpenoids from traditional Chinese plant *Flickingeria fimbriata* (B1.) Hawkes. Chin Chem Lett.

[15] Chen JL, Zhao ZM, Xue X, Tang GH, Zhu LP, Yang DP (2014). Bioactive norditerpenoids from *Flickingeria fimbriata*. RSC Adv.

[16] Wiens B, Luca VD (2016). Molecular and biochemical characterization of a benzenoid/phenylpropanoid meta/para-O-methyltransferase from *Rauwolfia serpentina* roots. Phytochemistry.

[17] Amen YM, Marzouk AM, Zaghloul MG, Afifi MS (2015). A new acylated flavonoid tetraglycoside with anti-inflammatory activity from Tipuana tipu leaves. Nat Prod Res.

[18] Le JM, Lu WQ, Xiong XJ, Wu ZJ, Chen WS (2015). Anti-inflammatory constituents from *Bidens frondosa*. Molecules.

[19] Żuchowski J, Pecio Ł, Stochmal A (2014). Novel flavonol glycosides from the aerial parts of lentil (*Lens culinaris*). Molecules.

[20] Li X, Tian Y, Wang T, Lin Q, Feng X, Jiang Q (2017). Role of the p-coumaroyl moiety in the antioxidant and cytoprotective effects of flavonoid glycosides: comparison of Astragalin and Tiliroside. Molecules.

[21] Stobiecki M (2000). Application of mass spectrometry for identication and structural studies of favonoid glycosides. Phytochemistry.

[22] Zhu H, Wang C, Qi Y, Song F, Liu Z, Liu S (2013). Fingerprint analysis of Radix Aconiti using ultra-performance liquid chromatography–electrospray ionization/tandem mass spectrometry (UPLC–ESI/MSn) combined with stoichiometry. Talanta.

[23] Cao J, Yin C, Qin Y, Cheng Z, Chen D (2014). Approach to the study of flavone di-C-glycosides by high performance liquid chromatography-tandem ion trap mass spectrometry and its application to characterization of flavonoid composition inViola yedoensis. J Mass Spectrom.

[24] Ferreres F, Andrade PB, Valentão P, Gil-Izquierdo A (2008). Further knowledge on barley (*Hordeum vulgare* L.) leaves O-glycosyl-C-glycosyl flavones by liquid chromatography-UV diode-array detection-electrospray ionisation mass spectrometry. J Chromatogr A.

[25] Swatsitang P, Tucker G, Robards K, Jardine D (2000). Isolation and identification of phenolic compounds in Citrus sinensis. Anal Chim Acta.

[26] Yang WZ, Qiao X, Bo T, Wang Q, Guo DA, Ye M (2014). Low energy induced homolytic fragmentation of flavonol 3-O-glycosides by negative electrospray ionization tandem mass spectrometry. Rapid Commun Mass Spectrom.

[27] Geng P, Sun J, Zhang M, Li X, Harnly JM, Chen P (2016). Comprehensive characterization of C-glycosyl flavones in wheat (*Triticum aestivum* L.) germ using UPLC-PDA-ESI/HRMS and mass defect filtering. J Mass Spectrom.

[28] Cuyckens F, Shahat AA, Van den Heuvel H, Abdel-Shafeek KA, El-Messiry MM, Nasr MMS-E (2003). The application of liquid chromatography-electrospray ionization mass spectrometry and collision-induced dissociation in the structural characterization of acylated flavonol O-glycosides from the seeds of *Carrichtera Annua*. Eur J Mass Spectrom.

[29] Ferreres F, Gil-Izquierdo A, Vinholes J, Grosso C, Valentão PC, Andrade P (2011). Approach to the study of C-glycosyl flavones acylated with aliphatic and aromatic acids from Spergularia rubra by high-performance liquid chromatography-photodiode array detection/electrospray ionization multi-stage mass spectrometry. Rapid Commun Mass Spectrom.

[30] Cuyckens F, Claeys M (2004). Mass spectrometry in the structural analysis of flavonoids. J Mass Spectrom.

[31] Wu CF, Gui SH, Huang YC, Dai YF, Shun QS, Huang KW (2016). Characteristic fingerprint analysis of *Dendrobium huoshanense* by ultra-high performance liquid chromatography-electrospray ionization-tandem mass spectrometry. Anal Methods.

[32] Zhang Y, Zhang L, Liu J, Liang J, Si J, Wu S (2017). *Dendrobium officinale* leaves as a new antioxidant source. J Funct Foods.

[33] Ferreres F, Silva BM, Ferreira MA (2003). Approach to the study of C-glycosyl flavones by ion trap HPLC-PAD-ESI/MS/MS: application to seeds of quince (*Cydonia oblonga*). Phytochem Anal.

